# Decolonization of methicillin-resistant *Staphylococcus aureus* – effectiveness of decolonization treatment

**DOI:** 10.1017/ash.2025.10070

**Published:** 2025-09-30

**Authors:** Tiina Haapia, Jaana Vuopio, Harri Marttila, Jaakko Silvola, Tero Vahlberg, Mari Kanerva, MRSA Study Group, Kirsi Gröndahl-Yli-Hannuksela, Anu Harttio-Nohteri, Jenna Junnila, Laura Lindholm, Kaisu Rantakokko-Jalava, Esa Rintala

**Affiliations:** 1 Infection Control Unit, Turku University Hospital, The Wellbeing Services County of Southwest Finland, Turku, Finland; 2 Institute of Biomedicine, University of Turku, Turku, Finland; 3 Clinical Microbiology Laboratory, Turku University Hospital, The Wellbeing Services County of Southwest Finland, Turku, Finland; 4 Finnish Institute for Health and Welfare, Helsinki, Finland; 5 Department of Biostatistics, University of Turku and Turku University Hospital, Turku, Finland

## Abstract

**Objectives:**

The aim of this study was to describe the efficacy of decolonization treatments given in Hospital District of Southwest Finland (HDSWF) in 2007–2016 and to analyze the key elements for successful decolonization treatment. Duration of follow-up varied from 12 to 15 months.

**Methods:**

All new MRSA cases detected between 2007 and 2016 in HDSWF (population 475,000) and their MRSA follow-up screening results within 12–15 months were retrospectively analyzed. This study focused on the outpatient carriers having received decolonization treatment during the study period.

**Results:**

Of the 983 MRSA cases detected during 2007–2016, 117 carriers went through decolonization treatment. Of those successfully followed up, 72/92 (78.3%) were successfully decolonized. Multisite carriage was a risk factor for unsuccessful decolonization.

**Conclusion:**

Decolonization treatment, including nasal mupirocin, chlorhexidine containing skin washes and in selected cases, also systemic antibiotics, was effective in outpatient settings, resulting in long-term clearance of the MRSA carriage.

## Background

Decolonization of methicillin-resistant *Staphylococcus aureus* (MRSA) carriage is recommended for selected carriers to prevent transmission in healthcare settings, but especially to prevent infection among carriers.^1,2^ The indications and methods vary in different settings and countries from universal search and destroy policy to prevent transmission and infections to tailored indications, for example prior to major surgery, to prevent surgical site infection.^2,3^

The pathways of spreading of MRSA in the community and the key elements to fight it are well established in the literature.^4–6^ There are national guidelines in Finland for the prevention of multidrug-resistant organisms, which outline active screening and contact precautions.^7^

In Finland, decolonization is offered to healthcare workers if persistent carriage is detected and to MRSA carriers prior to admission to a long-term care facility and rarely to carriers during hospitalization to prevent further transmissions.^7^ Also, patients suffering from recurrent skin infections and their families are being offered treatment to prevent further infections.^8^ However, all 21 hospital districts have their own, local guidelines giving more detailed instructions.

Even though decolonization treatments have been used for decades, the optimal treatment and follow-up time after the treatment still remain to be determined. Topical treatment is commonly used ^9^ and, especially if throat or multisite carriage is involved, systemic antibiotics are also given, since throat carriage has been acknowledged as one of the determinants for treatment failure.^10–12^ The added value of systemic treatment has, however, been discussed.^13^ In addition, the optimal duration and combination of antibiotic treatment are yet to be defined.^8,3^ The additional protocols, such as home cleaning, vary between countries.

In this study, we retrospectively analyzed the efficacy of decolonization protocols on individuals whose MRSA carriage was detected during a period from 2007 to 2016 in HDSWF. We also searched for the determinants of successful decolonization in order to learn who should be offered decolonization treatment.

## Methods

### Study setting

This registry-based retrospective study took place in HDSWF, which covers a population of 475,543 residents (year 2016),^14^ representing 8.6% of the total population in Finland (5.5 million).^15^ During the study period, 2007–2016, 983 new MRSA cases were detected in HDSWF either by clinical cultures (mainly abscesses, surgical site infections, infections of chronic wounds, etc.) or screening.^16^ Regarding MRSA, Finland was, and still is, a low-endemicity country.^17,18^ MRSA cases are usually detected via active risk-based screening on asymptomatic carriers.

### Risk-based MRSA screening

The screening policy included all direct hospital transfers from abroad, healthcare workers returning to work after working abroad, and hospitalized refugees. According to active contact tracing policy, all hospital contacts or roommates of a new MRSA case within the last six months were traced and their electronic health records were tagged with a screening alert to test them upon future admission. The screening sample set included swab samples from nose, throat, perineum, and possible colonization sites (eg, wounds, ulcers, catheters etc.) on two different days (> 24 h apart). Family members of the carriers were also screened if the index carrier was considered for decolonization treatment.

### Patients

The study population included those new MRSA carriers whose carriage was detected, and the decolonization treatment was given during the study period of 2007–2016.

### MRSA notification and registries

Finnish clinical microbiology laboratories notified all new MRSA findings by law from both clinical and screening samples to the National Infectious Disease Register (NIDR) maintained by the Finnish Institute for Health and Welfare (THL). The Turku University Hospital clinical microbiology laboratory performed MRSA diagnostics for HDSWF.^19^ The MRSA isolates were sent to the culture collection of NIDR. THL performed *spa* typing^20^ for them, and reported the result back to the hospital district. The *spa* types from 2007 to 2008 were analyzed at University of Turku.^16^ The strains were performed susceptibility testing.

### MRSA screening after decolonization treatment

**Figure 1.**
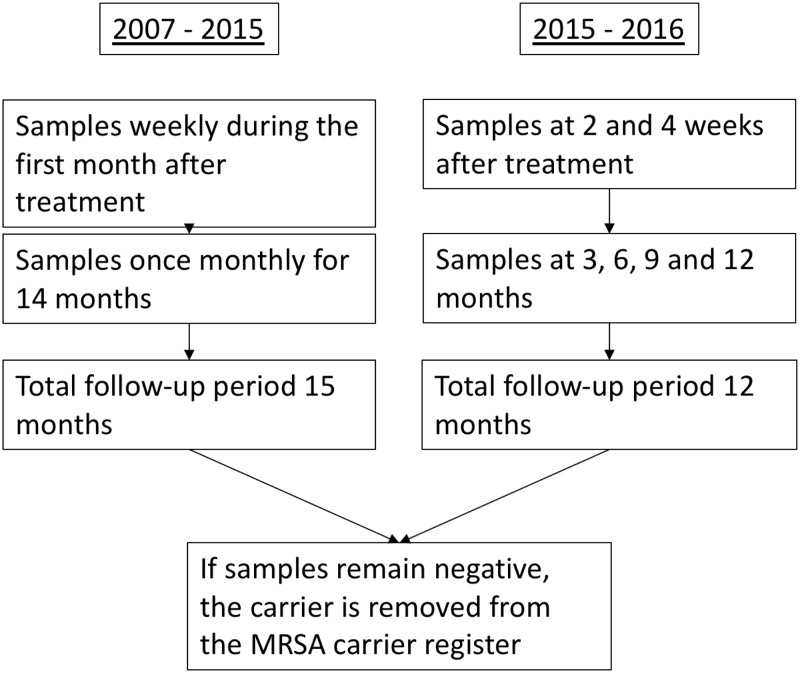
Follow-up after decolonization treatment.

MRSA screening samples were taken after decolonization treatment. The screening timetable is shown in Figure 1. Repetitive screening was ceased if the samples became positive. For persons with a relapse after decolonization treatment, a new treatment was offered.

### Patient data

Background information and medical history of patients were collected from electronic patient records. Medical conditions were classified using Charlson Comorbidity Index (CCI).^21^

### Detection of clearance of MRSA

Repetitive MRSA screening sample sets were taken after decolonization treatment (Figure 1) for 12–15 months, and if remained negative, the patient was considered having cleared MRSA carriage. If the samples turned positive during the follow-up period, a new treatment was offered.

### Decolonization treatment and regimens used

After a positive MRSA screening sample, a more thorough set of samples was taken in order to distinguish which body sites were colonized. Treatment was only considered for carriers whose samples were positive at least twice (taken 24 h apart). All decolonization treatments were centralized to the outpatient clinic in Turku University Hospital.

**Figure 2.**
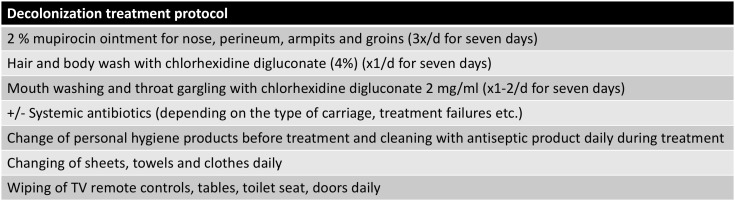
Decolonization protocol.

Indications for decolonization treatment varied but, in general, it was offered for healthcare workers, carriers suffering from recurrent infections, and their family members. The treatment always included topical treatment as well as thorough home cleaning. The treatment protocol is described in Figure 2. If the person was a throat carrier, systemic antibiotics were added to the treatment.

**Figure 3.**
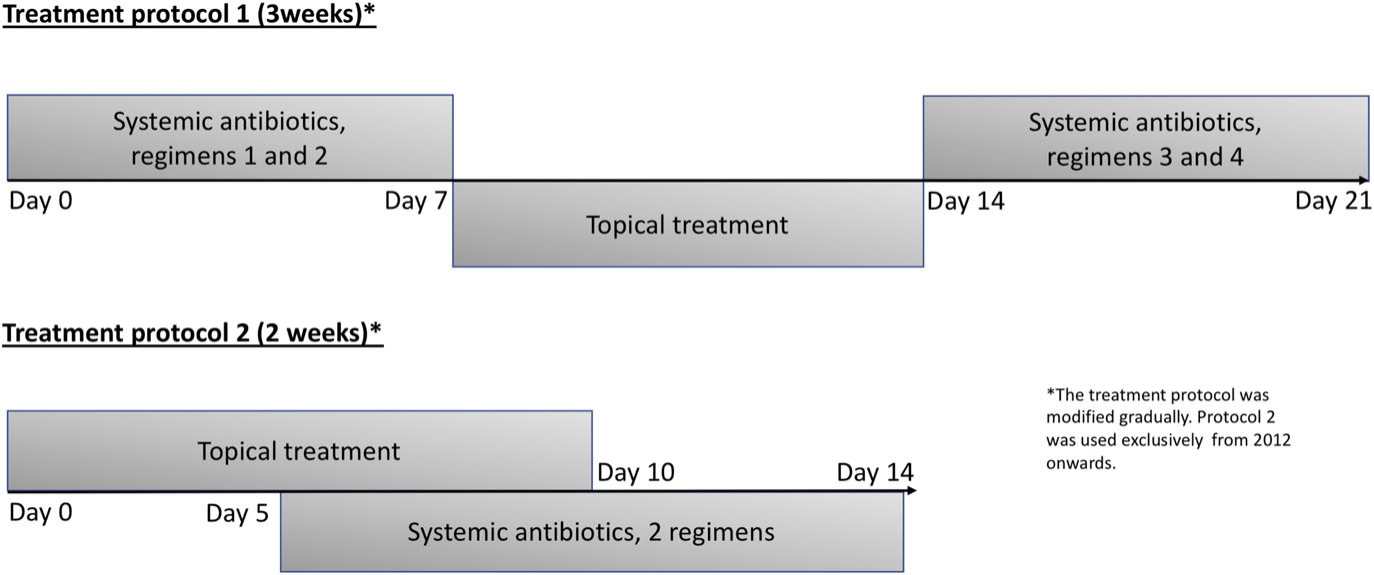
Treatment period, when systemic antibiotics were used.

Due to more recent research data, and change in national guidelines, the treatment protocol was slightly modified during the study period (Figure 3). From 2007 to 2011 the systemic treatment typically consisted of four different antibiotics (protocol 1). From 2012 onwards,^22^ only two different systemic antibiotics were given (protocol 2). The antibiotics were chosen based on the resistance profile of the MRSA strain in question, as no uniform policy regarding the selection of the antibiotics had been published. Also, possible antibiotic allergies or drug-drug interactions were taken into account. This resulted in a very heterogeneous setting of different antibiotics, but typically included at least one of the following: clindamycin, co-trimoxazole, fluoroquinolone or rifampin (supplement Table 2). No record was kept of possible side effects during or directly after the treatment, and they were only discovered, if the patient (or another treating physician, eg, family doctor) informed the outpatient clinic of any side effects.

### Follow-up

The follow-up period after the decolonization treatment varied from 12 to 15 months depending on the study period, Figure 1.

### Family clusters

Family clusters were defined as two or more MRSA carriers living in the same household.^16^

### Statistical analysis

The statistical analysis was made using IBM SPSS Statistics [version 28.1.0 (142)]. The determinants (eg, treatment type and duration, carriage type, number of carriage sites) for treatment failure were analyzed using binary logistic regression. Results are expressed using odds ratios (OR) with 95% confidence intervals (CI). *P*-values less than .05 were considered as statistically significant.

## Results

In total, 983 new MRSA cases were detected in HDSWF during 2007–2016 (annual range from 62 to 134 cases). Of these cases 280 (28%) were detected by clinical specimens and 703 (72%) by screening.

Of the 983 cases, 117 MRSA cases were given decolonization treatment during the study period.

Amongst those who were successfully followed up after decolonization treatment, 72/92 (78.3%) became MRSA negative, 61 of them (61/92, 66.3%) after the first treatment. One carrier was lost to follow-up after re-treatment. There were different reasons for losing patients to follow-up. Some might have moved to another HD during the follow-up period, and some might have been found to be positive during the follow-up period and was not for some reason considered for re-treatment.

The median age was 31.8 years (range 1–91 years). Patient characteristics and strain types are shown in Table 1. Their MRSA was detected mostly (83%) by screening, and they had a median of two sites colonized.

**Table 1.** Background information of the MRSA carriers and determinants for treatment failure

	Decolonization treatment given, N (%) N = 117	First decolonization successful, N (%) N = 61	First decolonization non-successful, N (%) N = 32	Lost to follow-up, N (%) N = 26	Univariate OR (95 % CI)	*p*-value	Multivariate OR (95% CI)	*p*-value
**Gender, female**	70 (59.8%)	38 (62.3%)	18 (56.3%)	15 (57.7%)	.78 (.33–1.86)	*p* = .572		
**Age**								
<18 years	19 (16.2%)	6 (9.8%)	7 (21.9%)	6 (23.1%)	ref			
18–64 years	91 (77.8%)	52 (85.2%)	22 (68.8%)	19 (73.1%)	.36 (.11–1.20)	*p* = .097		
>65 years	7 (6.0%)	3 (4.9%)	3 (9.4%)	1 (3.8%)	.86 (.12–5.94)	*p* = .876		
**Medical history (CCI points)**								
Medical history 0 or unknown	94 (80.3%)	46 (75.4%)	25 (87.1%)	24 (92.3%)	ref			
Medical history CCI 1–5 vs 0	19 (16.2%)	13 (21.3%)	5 (15.6%)	2 (7.7%)	.71 (.23–2.21)	*p* = .552		
Medical history CCI >5 vs 0	4 (3.4%)	2 (3.3%)	2 (6.3%)	0	1.84 (.24–13.86)	*p* = .554		
**Detection method**								
Detection by clinical sample	20 (17.1%)	9 (14.8%)	5 (15.6%)	6 (23.1%)	1.07 (.33–3.51)	*p* = .911		
Detection by screening	97 (82.9%)	52 (85.2%)	27 (84.4%)	20 (76.9%)	ref			
**Health care worker (HCW)**	38 (32.5%)	23 (37.7%)	6 (18.8%)	10 (38.5%)	.38 (.14–1.07)	*p* = .066		
**Belongs to a family cluster**	74 (63.2%)	33 (54.1%)	22 (68.8%)	20 (76.9%)	1.867 (.76–4.60)	*p* = .175		
**Carriage type^a^**								
Nasal only	9 (8.0%)	2 (3.4 %)	4 (12.5 %)	3 (11.5%)	4.39 (.76–25.48)	*p* = 1.000		
Nasal and other	63 (55.8%)	31 (52.5%)	20 (62.5%)	14 (53.8%)	1.81 (.72–4.51)	*p* = .205		
Throat only	13 (11.5%)	8 (13.6%)	1 (3.1 %)	4 (15.4%)	.22 (.03–1.85)	*p* = .163		
Throat and other	48 (42.5%)	31 (50.8%)	21 (65.6 %)	9 (34.6%)	4.55 (1.76–11.75)	*p* = .002	3.73 (1.39–10.00)	*p* = .009
Perineal only	5 (4.4%)	3 (5.1%)	0	2 (7.7%)				*
Perineal and other	43 (38.1%)	18 (30.5%)	17 (53.1 %)	9 (34.6%)	2.49 (1.04–5.97)	*p* = .040	2.10 (.79–5.57)	*p* = .135
Unknown	4 (3.4%)	2 (3.3%)	2 (6.3%)	0				
**Median number of sites colonized**	2 (IQR 2), range 1–7	2 (IQR 2), range 1–5	3 (IQR 3), range 1–7	1.5 (IQR 3), range 1–5	1.60 (1.14–2.25)	*p* = .006		
***spa* types**								
* spa* t172	44 (37.6%)	25 (41.0%)	12 (37.5%)	8 (30.8%)	.86 (.36–2.08)	*p* = .744		
* spa* t002	15 (12.8%)	6 (9.8%)	5 (15.6%)	4 (15.4%)	1.70 (.48–6.06)	*p* = .415		
* spa* t008	3 (2.6%)	1 (1.6%)	0	2 (7.7%)				*
* spa* t032	2 (1.7%)	1 (1.6%)	0	1 (3.8%)				*
Other *spa* types or not typed	53 (45.3%)	28 (45.9%)	15 (46.9%)	11 (42.3%)	1.04 (.44–2.45)	*p* = 1.040		
**Treatment type**								
Topical treatment	18 (15.4 %)	9 (14.8 %)	6 (18.8 %)	4 (15.4 %)	ref			
Systemic and topical treatment	97 (82.9%)	50 (82.0 %)	26 (81.3 %)	22 (84.6 %)	.78 (.25–2.43)	*p* = .668		
Other^b^	2 (1.7 %)	2 (3.3 %)	0	0				
**Rifampin-based systemic treatment**	74 (63.2%)	36 (59.0%)	20 (62.5%)	19 (73.1%)	1.16 (.48–2.79)	*p* = .744		
**Protocol**								
Protocol 1 (3 weeks)	31 (36.9%)	12 (11.1 %)	7 (31.8%)	12 (54.5%)	.78 (.25–2.43)	*p* = .668		
Protocol 2 (2 weeks)	53 (63.1%)	26 (24.1 %)	15 (68.2%)	5 (22.7%)	ref			
Other^c^	13 (13.4%)	9 (8.3%)	4 (8.0%)	5 (22.7%)				

^a^ Clinical sample marked as one carriage site.

^b^ Details not documented.

^c^ Tailored treatment period or details not documented.

* Fisher’s exact test. OR was not available due to zero frequency in non-successful group.

OR, odds ratio, Binary logistic regression. Patients with first decolonization (non-successful vs successful) were included in analyses (N = 93).

CI, confidence interval.

The first treatment for 97/117 carriers (82.9%) included systemic antibiotics (Figure 4, supplement), and for 18/117 carriers (15.4%) only topical treatment.

The first treatment failed in 32/117 (27.4%) carriers. Of these, six occurred after treatment with topical agents only and 26 following systemic treatment. The difference in failure rate was not statistically significant between the two different treatment protocols (topical vs systemic treatment, OR .78; 95% CI, .25–2.43, *p* = .668).

Anatomic carriage sites were determined in 30 of the treatment failures (Table 1). In univariate analysis, throat and perineal carriages were associated with treatment failure, when also other sites were colonized (Table 1), (OR, 4.55; 95% CI, 1.76 to 11.75, *p* = .002 and OR, 2.49; 95% CI, 1.04 to 5.97, *p* = .040, respectively), compared to other carriage sites. In multivariate analysis, only throat carriage together with other colonization sites was associated with treatment failure (OR, 3.73; 95% CI, 1.39–10.00, *p* = .009).

The number of carriage sites was also associated with treatment failure; the more carriage sites were detected, the more likely treatment was to fail (*p* = .006). In successfully treated carriers, median (interquartile range) carriage sites were 2 (2) and in treatment failures 3 (2) (Table 1).

Regarding treatment failures, there was no statistically significant difference between *spa* types or whether MRSA was originally detected by screening or from a clinical sample (Table 1).

Retreatment was given to 19/117 carriers, all systemic treatments. Altogether 11 of them were successfully retreated. Two carriers received three treatments, and still remained MRSA positive. They did not belong to a same family cluster nor had same *spa* types.

The duration of treatment varied during the study period. Altogether 31/117 carriers (26.5%) received a three-week treatment (protocol 1), and 53/117 carriers (45.3%) received a two-week treatment (protocol 2). For 13 carriers (11.1%), the treatment duration was modified. Of protocol 1, 19/31 (61.3%) carriers and of protocol 2, 27/53 (50.9%) carriers became negative after the first treatment. In univariate analysis, there was no statistically significant difference between protocols in treatment failure (OR, .78; 95% CI, .25–2.43, *p* = .668) (Table 1).

There were no statistically significant differences in the efficacy of rifampin versus non-rifampin based antibiotic combinations given within the systemic treatment group. (Table 1).

Adverse events were recorded for only six carriers (6/117, 5.1%), all having received systemic treatment. Three were *Clostridioides difficile* infections, all mild, treated with metronidazole. Hypertension was reported in one carrier while receiving decolonization with rifampin. One carrier suffered from thrush. In one case the adverse events were not specified.

### Healthcare workers

Decolonization treatment was given to 38 healthcare workers, 29 of them were successfully followed up. Of these, 23/29, (79.3%) became negative after the first treatment. Six failed the first treatment (five throat and one nasal carriers). Second treatment was given to 5/6 treatment failures, with systemic antibiotics. Three became negative after the second treatment, one was lost to follow-up at this point. The total success rate in this group was 92.9% (26/28). The carrier characteristics are shown in Table 1.

## Discussion

Decolonization treatment was effective, the success rate being 78.3%, and among healthcare workers as high as 92.9%. Compared to many other studies ^9,10^, we had a long follow-up period, which resulted in losing some carriers to follow-up, but better demonstrated long-term efficacy of the treatment.

We did not find differences between the two- and three-week treatment protocols nor between topical and systemic treatments. The latter may, however, be due to careful selection of treatment type based on the anatomic carriage profile. In line with a study by Bagge et al,^9^ our findings also showed that throat and perineal carriage are risks for treatment failure. The increasing number of carriage sites was also a statistically significant risk for failure. In the future, it might be reasonable to consider a treatment protocol that is also based on the number of carriage sites.

We did not find differences in the efficacy between the different antibiotic combinations given. This may indicate that, as treatment regimens were selected based on the susceptibility of the strain, the failure was due to reasons other than antibiotics not having been effective. The compliance of the patients to the treatment protocol was not recorded. The treatment protocol is quite exhausting, and possible lack of compliance could have some role in success rates. Whether for example patient adherence varies in different age groups, is an issue needing further studies.

Moreover, we could not show any higher efficacy with rifampin containing regimens either, although there are reports indicating that these might be superior.^23^ Westgeest et al had similar findings in their retrospective study.^24^ The optimal treatment regimen(s) and duration of treatment still remain an area of interest, and it would need a prospective study to further evaluate whether different treatment methods would reveal statistically significant differences.

Although adverse events with antibiotics are fairly common^25^, we found documentation of these only in five per cent of the patients, and no major adverse events were found. We did not collect these reactions systematically, however, and cannot exclude all possible events. For a number of reasons, it would be reasonable to use courses of antibiotics that are as few and as short as possible.

The strength of the study is that it is population-based, and decolonization treatment has been systematically described and performed. Follow-up is organized uniformly in the clinical care of the patient. However, some patients opted out of follow-up. Furthermore, in Finland it is mandatory to notify THL of all new MRSA cases, resulting in a very reliable record on all MRSA carriers. We also actively screen for MRSA carriage in Finland. The local electronic patient record systems allow for convenient access to information and facilitated subsequent data analysis.

There are some limitations to this study. Firstly, the study was retrospective, and secondly the data was collected from the hospital healthcare records and not by interviews. Moreover, we did not have access to the majority of documentation concerning primary or private care visits, did not have data on patients’ compliance to the treatment protocol, the possible clinical infections occurring after the follow-up period, or the possible existence of mupirocin resistance.

## Conclusions

Our findings suggest that decolonization treatment can be effective, if the carriers who are offered treatment are carefully selected and when multisite carriage is treated with systemic antibiotics in addition to topical treatment. Treatment failures are associated with multisite colonization, including either throat or perineum carriage.

## Supporting information

10.1017/ash.2025.10070.sm001 Haapia et al. supplementary material 1 Haapia et al. supplementary material

10.1017/ash.2025.10070.sm002 Haapia et al. supplementary material 2 Haapia et al. supplementary material

## Data Availability

The datasets generated during the current study are not publicly available as they contain health-related data, but limited datasets (without any identifiable, person-related data) are available from the corresponding author on reasonable request.

## Supplementary material

The supplementary material for this article can be found at https://doi.org/10.1017/ash.2025.10070.
