# Decolonization of methicillin-resistant *Staphylococcus aureus* – effectiveness of decolonization treatment — ADDENDUM

**DOI:** 10.1017/ash.2025.10226

**Published:** 2025-12-17

**Authors:** Tiina Haapia, Jaana Vuopio, Harri Marttila, Jaakko Silvola, Tero Vahlberg, Mari Kanerva, MRSA Study Group

In the original publication of this article,^1^ the names of the individual members of the MRSA Study Group were omitted from the list of authors.

The article has been updated to include this information.
