# Supplementary figures and images for: Decolonization of methicillin-resistant Staphylococcus aureus – effectiveness of decolonization treatment
Source: Antimicrob Steward Healthc Epidemiol. 2025 Sep 30;5(1):e234. doi: 10.1017/ash.2025.10070 (PMC12509158; doi:10.1017/ash.2025.10070)

Antibiotics given for the first decolonization treatment

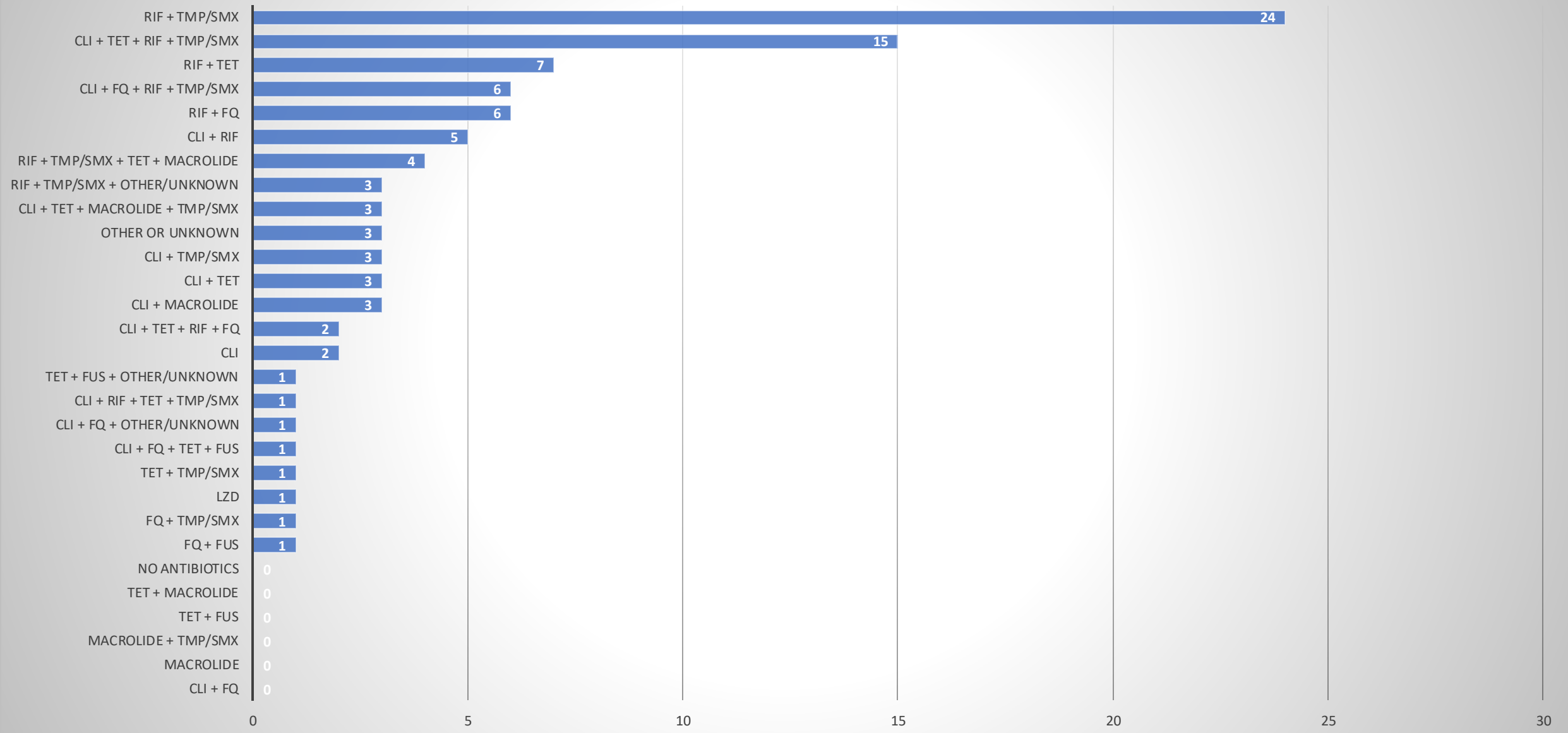

Supplement: Haapia et al. supplementary material 1 — Haapia et al. supplementary material [file S2732494X25100703sup001.pdf]
